# A systematic evaluation of high-dimensional, ensemble-based regression for exploring large model spaces in microbiome analyses

**DOI:** 10.1186/s12859-015-0467-6

**Published:** 2015-02-01

**Authors:** Jyoti Shankar, Sebastian Szpakowski, Norma V Solis, Stephanie Mounaud, Hong Liu, Liliana Losada, William C Nierman, Scott G Filler

**Affiliations:** 1 J. Craig Venter Institute, 9704, Medical Center Drive, Rockville, Maryland, 20850 US; 2 Los Angeles Biomedical Research Institute at Harbor, UCLA Medical Center, 1124 West Carson Street, Torrance, California, 90509 US; 3 David Geffen School of Medicine, University of California at Los Angeles, California, 90095 US

**Keywords:** Microbiome analysis, Sparse ensemble-based regression, Simulation design, Stability selection, Bayesian model averaging, Evaluation

## Abstract

**Background:**

Microbiome studies incorporate next-generation sequencing to obtain profiles of microbial communities. Data generated from these experiments are high-dimensional with a rich correlation structure but modest sample sizes. A statistical model that utilizes these microbiome profiles to explain a clinical or biological endpoint needs to tackle high-dimensionality resulting from the very large space of variable configurations. Ensemble models are a class of approaches that can address high-dimensionality by aggregating information across large model spaces. Although such models are popular in fields as diverse as economics and genetics, their performance on microbiome data has been largely unexplored.

**Results:**

We developed a simulation framework that accurately captures the constraints of experimental microbiome data. Using this setup, we systematically evaluated a selection of both frequentist and Bayesian regression modeling ensembles. These are represented by variants of stability selection in conjunction with elastic net and spike-and-slab Bayesian model averaging (BMA), respectively. BMA ensembles that explore a larger space of models relative to stability selection variants performed better and had lower variability across simulations. However, stability selection ensembles were able to match the performance of BMA in scenarios of low sparsity where several variables had large regression coefficients.

**Conclusions:**

Given a microbiome dataset of interest, we present a methodology to generate simulated data that closely mimics its characteristics in a manner that enables meaningful evaluation of analytical strategies. Our evaluation demonstrates that the largest ensembles yield the strongest performance on microbiome data with modest sample sizes and high-dimensional measurements. We also demonstrate the ability of these ensembles to identify microbiome signatures that are associated with opportunistic *Candida albicans* colonization during antibiotic exposure. As the focus of microbiome research evolves from pilot to translational studies, we anticipate that our strategy will aid investigators in making evaluation-based decisions for selecting appropriate analytical methods.

**Electronic supplementary material:**

The online version of this article (doi:10.1186/s12859-015-0467-6) contains supplementary material, which is available to authorized users.

## Background

Biological experiments that incorporate extensive next-generation sequencing (NGS) to profile microbial communities are becoming increasingly common in the field of medical microbiology and elsewhere [1-3]. In a substantial number of these studies, the primary objective is to establish associations between the microbial community and specific biological traits or endpoints of clinical relevance. Analytical methods to test these associations follow three general directions. The first explains microbial abundance profiles in terms of biological and clinical variables [4-11]. The second utilizes these microbial profiles to classify samples into biological or clinical categories (e.g., human body sites, health and disease states, subject identities, etc.) [12-15]. A third explains a clinical or biological endpoint in terms of microbial community profiles in combination with other biological covariables [16,17]. The first two directions have formed the basis for most of the analytical work in this field. In this work, we explore the third direction.

**Figure 1:**
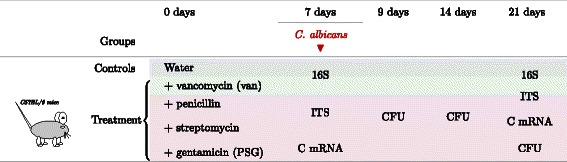
**Experimental design of the mouse microbiome study.** Mice were divided into a control group and two treatment groups. The treatment groups received either vancomycin (van) or a combination of penicillin, streptomycin and gentamicin (PSG). After 7 days, some of the mice were administered a *C. albicans* challenge. Terminal ileum samples were collected on days 7 and 21 and fecal pellet samples on days 7, 9, 14 and 21. *C. albicans* colonization level was measured by counting the Colony forming units (CFU) following quantitative culture of the fecal pellets. Cytokine mRNA expression (C mRNA) was measured in the terminal ileum samples. Bacterial 16S and Fungal ITS were amplified from both the terminal ileum sections and the fecal pellets. These amplicons were sequenced with 454 pyrosequencing. The YAP bioinformatic workflow [54] was used to obtain genus-level taxonomic assignments for the bacterial and fungal sequences.

Specifically, we used a mouse model to investigate the risk factors during antibiotic therapy for opportunistic, gastrointestinal (GI) colonization with *Candida albicans*, a commensal fungus in the human GI tract (Figure 1 and Table 1). *C. albicans* does not normally colonize mice unless the resident GI flora is perturbed by antibiotics. Therefore, we exposed mice to a single challenge with *C. albicans* 7 days after initiating antibiotic treatments that spanned a total of 21 days. Our initial observations indicated that some antibiotics induced a persistently higher level of *C. albicans* colonization compared to others. We sought further insights into these differential patterns of colonization by identifying the underlying antibiotic-induced perturbations in the bacterial and fungal GI microbiome, and the host immune factors. Hence, we employed a regression framework to explain the level of *C. albicans* colonization using microbiome variables, immune factors and experimental conditions as covariables.

**Table 1:** **Characteristics of data from the mouse microbiome study**

**Measurement**	**Original variable**	**Pre-processing**	**Dimension**	**Role in models**
16S	Sequence counts	*log* (Relative abundances)	344	Independent
ITS	Sequence counts	*log* (Relative abundances)	109	Independent
C mRNA	Expression levels	*log* (GAPDH normalized expression levels)	6	Independent
CFU	Colony counts	$log \left (\frac {\text {Colony forming units (CFU)}}{\text {grams of fecal matter}}\right)$	1	Dependent (Response)
Antibiotic treatment				
±*C. albicans* challenge				
+ Length of treatment	Categorical	Dummy encoding	5 levels	Independent

*C. albicans* colonization level was measured by counting the Colony forming units (CFU) following quantitative culture of the fecal pellets. Cytokine mRNA expression (C mRNA) was measured in the terminal ileum samples. Bacterial 16S and Fungal ITS were amplified from both the terminal ileum sections and the fecal pellets. These amplicons were then sequenced by 454 pyrosequencing and taxonomically classified with a tailored bioinformatic pipeline [54].

We faced unique challenges in performing these analyses. Our studies utilized animal models with tens of samples (*n*). However, the total number of variables (*p*) arising from NGS measurements of the microbiome, host immune factors and other biological exposures was at least an order of magnitude larger than the number of samples (*p*>>*n*). In this scenario, a model estimated using univariate or non-regularized regression modeling strategies [6,8] is likely to overfit the data and yield findings that would not generalize well on data from newer studies [18]. Moreover, this model does not take into account the covariance structure of high-dimensional data, making the naïve assumption of orthogonality of covariables. In addition, such a model is only one among the 2^*p*^ possible model configurations that could explain the data [19,20]. We therefore needed a modeling strategy which a) incorporates penalized regression to avoid overfitting, b) addresses multicollinearity, c) efficiently searches through a large number of model configurations prior to settling on the most likely model specification, and d) estimates the model parameters with low variability in the face of modest sample sizes.

Ensemble models are a class of approaches that elegantly satisfy all of the above requirements by merging information on the explanatory variables from a large collection of models to generate a ranked list of influential variables ordered by their importance [20]. These ensembles estimate robust models with stable solutions by utilizing the inherent variability in either the sample space through bootstrap methods [21,22] or the model space using Bayesian methods [23] and Markov chain Monte Carlo (MCMC) algorithms [24].

Random forests [25], a non-linear ensemble method, has been widely used for classification and prediction tasks in microbiome research [12,14]. However, random forests are not directly usable in a problem domain such as ours that requires the estimation of a model in a linear regression framework for a continuous clinical endpoint of interest. Investigators in the field have adopted penalized regression methods [4,5,14-16] that are able to effectively model multivariable covariances within the microbiome. Nevertheless, these methods have been employed in a non-ensemble setting which could potentially lead to instability in model findings.

Only a few research groups have incorporated ensemble linear regression modeling in a problem domain similar to ours [17]. Furthermore, there has been no assessment of how the characteristics of linear regression ensembles affect model performance on realistic microbiome data. We address this gap in the microbiome literature by a) rigorously evaluating the performance of a selection of frequentist and Bayesian ensemble regression modeling approaches, and b) introducing a simulation and evaluation framework that can be constructed to closely mimic the characteristics of any experimental microbiome dataset. Using this framework and a suite of metrics, we determine how various characteristics of ensembles influence model performance. We also demonstrate the application of these ensembles to our experimental mouse data. Results from our evaluation demonstrate that ensembles capable of exploring larger model spaces perform better with lower variability.

## Methods

### Simulation design

We generated data for our simulation study using the linear model,

(1) $$ Y = \boldsymbol{X}\boldsymbol{\beta} + \boldsymbol{\epsilon},  $$

where *Y* is the response variable, ***X*** is the design matrix with *n* samples and *p* variables, ***β*** is the p-dimensional vector of regression coefficients and the random error $\boldsymbol {\epsilon } \sim \mathcal {N}\left (0, \sigma ^{2} \cdot \text {I}_{n}\right)$ where I_*n*_ is the identity matrix. We describe the construction of the components ***X***, ***β*** and ***ε*** below.

#### Design matrices

We constructed two design matrices based on the experimental mouse gut bacterial and fungal microbiome profiles, respectively. We first eliminated the rows and columns which had zero counts across all samples; *n* and *p* refer to the total number of rows and columns that remained. In the bacterial matrix, *n*=30 and *p*=192 while in the fungal matrix, *n*=36 and *p*=47. We then constructed the design matrix ***X*** by log-transforming the relative abundances of genera, $t^{\prime }_{\textit {ij}}$ where *i*∈{1,2,…*n*} and *j*∈{1,2,…,*p*}.

(2) $$ \phi_{ij} = \left(\frac{t_{ij} + 1} {\sum_{j=1}^{p} t_{ij} + 1} \right), t^{\prime}_{ij} = log(\phi_{ij}),  $$

where *ϕ*_*ij*_ and *t*_*ij*_ denote the relative abundance and the sequence count of bacterial or fungal genus, *j* in sample *i*, respectively. We added a smoothing constant of 1 to *t*_*ij*_ to ensure non-zero proportions for all genera. The log-transformation ensures that all variables have a similar dynamic range across all the samples [26]. *p* denotes the total number of genera.

Since $\sum _{j=1}^{p} \phi _{\textit {ij}} = 1$ for every *i*, the relative abundance of one of the genera is fully specified by the others. To address this redundancy, we selected one of the genera as a reference, and dropped it from the design matrix ***X***. We chose the reference genus, *j*_ref_ to be the one with the greatest abundance across all the samples. i.e.,

(3) $$ j_{\text{ref}} = \mathop{\text{argmax}}\limits_{j \in \{1,2,\ldots\,p\}} \sum\limits_{i=1}^{n} t_{ij}.  $$

#### Influential variables

Microbiome sequence data describes an entire community of organisms. It is not known whether this community consists of a large or a small subset of influential microbes. In addition, their degree of association with the response is also unknown. We addressed this uncertainty using the following strategies: a) considering a range of sparsity settings (% of variables specified as influential ∈{2*%*,3*%*,4*%*}), and b) sampling regression coefficients from either a bimodal distribution, *β*=±1 or a uniform distribution, $\beta \in \mathcal {U}$.

We denote the set of specified influential variables as $\mathcal {V}_{i}$. We randomly selected the indices of these variables and divided them into two halves $\mathcal {V}_{i}^{1}$ and $\mathcal {V}_{i}^{2}$. We used two alternative schemes for assigning regression coefficients ***β*** to the selected variables in $\mathcal {V}_{i}^{1}$ and $\mathcal {V}_{i}^{2}$, following the approach in Meinshausen et al. [22].

1. *β*=±1: $\forall v \in \mathcal {V}_{i}^{1}, \beta _{v}=1; \: \forall v \in \mathcal {V}_{i}^{2}, \beta _{v}=-1$.
2. $\beta \in \mathcal {U}$: $\forall v \in \mathcal {V}_{i}^{1}, \beta _{v} \sim \mathcal {U}(0.5, 1.0); \: \forall v \in \mathcal {V}_{i}^{2}, \beta _{v} \sim \mathcal {U}(-1.0,-0.5)$.

#### Signal to noise ratio

The signal to noise ratio (SNR) is defined as [27]

(4) $$ \text{SNR} = \frac{||\boldsymbol{X}\boldsymbol{\beta}||_{2}}{\sqrt{n} \sigma},  $$

where ||***X*** ***β***||_2_ is the *l*_2_ norm of the predicted response. We computed the SNRs in our experimental mouse data by estimating an elastic net model [28]. The bacterial data had an SNR of 4.6, while the fungal data had an SNR of 5.2. In our simulation, we applied three different SNRs of 0.25, 4.60 and 16.00, that correspond to high, medium, and low noise settings, respectively. For a given SNR, we sampled the random error $\epsilon _{i} \sim \mathcal {N}(0, \sigma ^{2})$ where *i*∈{1,2,…*n*} and where *σ* is chosen as:

(5) $$ \sigma = \frac{||\boldsymbol{X}\boldsymbol{\beta}||_{2}}{\sqrt{n} \cdot \text{SNR}}.  $$

It is easier to recover the influential variables under a higher SNR since the response has a larger association with the variables relative to the random error term.

For each combination of ***X***, ***β*** and SNR, we performed a total of 130 simulations and report results aggregated over these runs, making our findings robust to variations. We conducted our evaluation and analysis in the R language for statistical computing [29] and have released our code at *GitHub* [30].

### Modeling approaches

In our evaluation, elastic net with cross-validation (ENC) was the baseline non-ensemble penalized regression method. We chose the remaining frequentist and Bayesian ensemble modeling approaches with the objective of determining how the size of the ensemble, construction of the model space, and the choice of regularization parameters within the ensemble influenced performance. We summarize these approaches in Table 2 and describe them in detail below.

**Table 2:** **Summary of the modeling approaches included in the evaluation**

**Model**	**Ensemble characteristics**		**Output**	**Paradigm**	**R Package**
	**Tuning parameter**	**Model space construction**			
ENC	*λ*_*ENC*_	None	Influential variables	$\left.\vphantom {\frac {\frac {\sum \limits ^{\frac {\sum \limits ^{2}_{3}} {1}}_{1}}{\sum \limits ^{2}_{3}}}{1_{3}^{4}}}\right \}\text {Frequentist}\ (l_{1}, l_{2}\ \text {penalties})$	*quadrupen, glmnet*
PS	*λ*_*MB*_	$\left.\vphantom {\frac {\sum \limits ^{2}_{3}}{1_{1}^{\frac {1}{2}}}}\text {Subsampling}\right \}$	Influential variables		
LS	*λ*_*ENC*_		Inclusion probabilities		
SS	*Λ*		Inclusion probabilities		
PR	*λ*_*MB*_	$\left.\vphantom {\frac {\sum \limits ^{2}_{3}}{1_{1}^{\frac {1}{2}}}}\right \}\text {Resampling}$	Influential variables		
LR	*λ*_*ENC*_		Inclusion probabilities		
SR	*Λ*		Inclusion probabilities		
BMA	*E* *M* *S*=1	$\left.\vphantom {\frac {\sum \limits ^{2}}{1_{3}}}\right \}\text {MCMC}$	Inclusion probabilities	$\left.\vphantom {\frac {\sum \limits ^{2}}{1_{3}}}\right \}\text {Bayesian (Spike \& slab prior)}$	*BoomSpikeSlab*
BMAC	*E* *M* *S*_*CV*_		Inclusion probabilities		

**ENC:** The baseline penalized regression model. Elastic net with *λ*_*optimal*_=*λ*_*ENC*_ derived from cross-validation (CV), **Ensembles based on 100 subsamples:** **PS:** Meinshausen & Bühlmann’s algorithm with a single *λ*_*optimal*_=*λ*_*MB*_ selected to minimize the expected number of false positives, **LS:** Single *λ*_*optimal*_=*λ*_*ENC*_ with no variable selection, **SS:** Stability selection across the entire 100 *λ*∈*Λ* grid with no variable selection, **Ensembles based on 100 resamples:** **PR, LR, SR:** Identical to PS, PR and LR, respectively, with model space constructed through resampling. **BMA:** Bayesian model averaging with expected model size (*EMS*) = 1, **BMAC:** BMA with EMS determined by CV (*E* *M* *S*_*CV*_).

#### Elastic net with cross-validation (ENC)

The ENC procedure [28] is a specific instance of a penalized regression model [31,32].

(6) $$ \hat{\boldsymbol{\beta}} \,=\, \mathop{\text{argmax}}\limits_{\boldsymbol{\beta}} \!\left\{\! \sum\limits_{i=1}^{n} |y_{i} \,-\, X_{i}\boldsymbol{\beta}|^{2} \,+\, \lambda\!\left(\!\alpha ||\boldsymbol{\beta}||_{1} \,+\, (1-\alpha)\!\frac{1}{2}\!||\boldsymbol{\beta}||^{2}_{2}\!\right) \!\right\}\!,  $$

where $\hat {\boldsymbol {\beta }}$ is the solution to the estimation problem, the penalty terms, $\|\boldsymbol {\beta }\|_{1} = \sum _{i=1}^{p} |\boldsymbol {\beta }_{i}|$ and $\|\boldsymbol {\beta }\|_{2} = \sqrt {\sum _{i=1}^{p} |\boldsymbol {\beta }_{i}|^{2}}$ are the *l*_1_ and *l*_2_ norms of the coefficient vector ***β***, $\sum _{i=1}^{n} |y_{i} - X_{i}\boldsymbol {\beta }|^{2}$ is the residual sum of squares (RSS), *λ* is the tuning parameter that penalizes the RSS by the size of the regression coefficients and *α* is a tuning parameter that balances the *l*_1_ and *l*_2_ penalties. An *α* of 1 promotes sparsity in the model, while an *α* of 0 ensures that correlated variables are assigned similar regression coefficients. An optimal value of *α* finds a balance between the two penalties. We employed leave-one-out cross-validation to determine the optimal combination of *α* and *λ* (*λ*_*optimal*_=*λ*_*ENC*_) that minimized the mean squared error (MSE) of the model [31]. We used the implementation of ENC available in the *glmnet* R package [32].

#### Frequentist ensembles

We evaluated frequentist ensembles that were variations on the ensemble strategy originally described by Meinshausen and Bühlmann (MB) [22]. In their work, MB proposed a method to improve stability of variable selection within the least absolute shrinkage and selection operator (LASSO) approach [33]. Since ENC is a generalization of the LASSO approach and our baseline, we replaced LASSO with ENC in MB’s stability selection procedure.

##### Ensembles based on subsamples (PS, LS, SS, SSW)

Within MB’s stability selection procedure, we first generated 100 subsamples of the data. From each subsample, we estimated regression coefficients ***β*** on a grid of regularization parameters *λ*∈*Λ*. *Λ* consisted of 100 *λ* values obtained using the Least angle regression (LARS) algorithm within ENC [31,34] implemented in the *glmnet* R package [32]. Given the set of selected variables at any *λ*, *S*(*λ*)={*j*:*β*_*j*_(*λ*)≠0}, the inclusion probability *P*(*j*∈*S*(*λ*)) of any variable *j*∈{1,2,…*p*} was computed as

(7) $$ P(j \in S(\lambda))= \frac{1}{B} \sum\limits_{b=1}^{B} 1_{j \in S_{\lambda}(b)},  $$

where *b* is the index of any given subsample, *B*=100 is the total number of subsamples and *S*_*λ*_(*b*) is the set of selected variables at regularization parameter *λ* in subsample *b*. To minimize the expected number of false positives within the selected set of variables, *S*(*λ*), MB developed an algorithm to select both the optimal regularization parameter *λ*_*optimal*_=*λ*_*MB*_ and a stable set of variables, $\hat {S}^{stable} \subseteq S(\lambda _{\textit {MB}})$ [22]. We adopted an implementation of MB’s algorithm from the *quadrupen* R package [35]. Since MB’s algorithm was designed to reduce false positives and employed subsampling to construct the model space, we called this modeling approach, PS. In addition to PS, we evaluated ensemble variants which enabled us to determine how performance was influenced by the choice of the regularization parameter *λ*_*optimal*_, size and nature of the model space, and the computation strategy for inclusion probabilities.

In the first variant of PS, we chose the single regularization parameter *λ*_*optimal*_=*λ*_*ENC*_, specified by the ENC procedure in *glmnet* [32]. Unlike PS that performed variable selection at *λ*_*MB*_, we computed the inclusion probabilities of the *p* variables across the 100 subsamples at *λ*_*ENC*_ using Equation 7. We termed this variant LS since it operated at a single *λ*_*optimal*_ and employed subsampling to construct the model space.

In the next variant, we expanded the size of the model space by computing the average inclusion probabilities, *I*_*j*_, of the variables across the 100 regularization parameters *λ*∈*Λ*,

(8) $$ I_{j} = \frac{1}{100} \cdot \sum\limits_{i = 1}^{100} P(j \in S(\lambda_{i})),  $$

where *j*∈{1,2,…*p*}. The approach assigned high inclusion probabilities to the stable variables which were most consistently selected across the *Λ* grid and employed subsampling to construct the model space. Hence we termed this variant SS.

We also considered an alternative definition of inclusion probability, ${I}^{w}_{j}, j \in \{1,2,\ldots,p\}$ based on a suggestion from our referee.

(9) $$ {I^{w}_{j}} = \sum\limits_{i = 1}^{100} w_{\lambda_{i}} \cdot P(j \in S(\lambda_{i})),  $$

where $w_{\lambda _{i}}$, the weight assigned to *λ*_*i*_, is proportional to the average number of non-zero variables at *λ*_*i*_

(10) $$ w_{\lambda_{i}} = \frac{{\sum\nolimits}_{b=1}^{B} 1_{j \in S_{\lambda_{i}}(b)}}{ {\sum\nolimits}_{i=1}^{100} {\sum\nolimits}_{b=1}^{B} 1_{j \in S_{\lambda_{i}}(b)}},  $$

where *b* is the index of the subsamples and *B* = 100 is the total number of subsamples. We termed this variant SSW.

##### Ensembles based on resamples (PR, LR, SR, SRW)

Since MB [22,27] recommend the use of resampling as an equivalent alternative to subsampling in the context of stability selection, we varied the nature of model space construction within the frequentist ensembles by adopting bootstrap resampling as an alternative to subsampling with size $\lfloor \frac {n}{2}\rfloor $. Subsampling without replacement and sampling at random with replacement (bootstrap resampling) are examples of schemes within a family of *exchangeably weighted bootstrap schemes* [36,37]. The equivalence of the two bootstrapping schemes has been described in [38] and [39] and demonstrated through theoretical results and extensive simulations of non-linear regression [40]. In addition, the two schemes have very similar statistical properties [41]. It has also been independently shown that bootstrap resampling improves stability of the LASSO procedure, which is a special case of ENC [42].

We implemented the resampling alternatives to PS, LS, SS and SSW, and termed them PR, LR, SR and SRW, respectively.

#### Bayesian ensembles

In addition to the frequentist ensembles, we evaluated Bayesian ensembles that further expanded the size of the model space explored. These larger ensembles were based on spike-and-slab Bayesian model averaging (BMA) [24] implemented in the *BoomSpikeSlab* R package [43]. Given a set of multicollinear variables, spike-and-slab BMA assigns higher inclusion probabilities to influential variables highly associated with the response. Strongly correlated variables that are equally associated with the response are assigned similar inclusion probabilities. Thus the BMA framework is conceptually similar to our baseline frequentist penalized regression approach, ENC, and its ensemble variants.

The spike-and-slab prior consists of two components and is described in detail in [44,45]. The spike component of the prior is modeled by a Bernoulli distribution for each variable and is described by

(11) $$ {\boldsymbol{\gamma}} \sim \prod\limits_{i=1}^{p} \pi_{i}^{(\gamma_{i})} (1- \pi_{i})^{(1-\gamma_{i})},  $$

where ***γ*** is a vector of binary indicators, *γ*_*i*_;*i*∈{1,2…*p*}. If *γ*_*i*_=1, the *i* ^*th*^ variable is selected as influential. If *γ*_*i*_=0, the *i* ^*th*^ variable is not influential and *β*_*i*_=0. *π*_*i*_ is the probability of the *i* ^*th*^ variable being influential and computed as $\pi _{i}=\frac {k}{p}$. *k* is the expected model size and specifies the number of variable coefficients expected to be non-zero.

Conditional on the set of influential variables {*i*:*γ*_*i*_=1}, the slab component of the prior distribution models the regression coefficients ***β*** for these variables. The distribution is a variant of the Zellner’s *g*-prior [46] and is given by

(12) $$ {\boldsymbol \beta} | \left(\sigma^{2}, {\boldsymbol \gamma} \right) \sim \mathcal{N} \left({\boldsymbol b}, \boldsymbol{F_{\gamma}}^{-1}\right),  $$

where ***b*** denotes the prior expectation of ***β*** and is set to ***0*** in our experiments. ***F***_***γ***_ is the sub-matrix that corresponds to the influential variables {*i*:*γ*_*i*_=1} within the full prior Fisher information matrix,

(13) $$ \boldsymbol{F} = \frac{g}{n} \cdot \frac{\boldsymbol{X^{T} X}}{\sigma^{2}}.  $$

In the event of multicollinearity, ***F*** may not always be positive definite. Therefore, the BMA implementation within *BoomSpikeSlab* ensures a proper posterior distribution by linearly interpolating ***X*** ^***T***^ ***X*** with its diagonal to obtain the smoothed Fisher information matrix [44,45],

(14) $$ \boldsymbol{F}_{smooth} = \frac{g}{n} \cdot \frac{w \times \boldsymbol{X^{T} X} + (1-w) \times \text{diag}(\boldsymbol{X^{T} X})}{\sigma^{2}},  $$

where *g* is set to 1 and *w*=0.5. *σ* ^2^ is the variance of the random error ***ε*** in the regression model and distributed as

(15) $$ \frac{1}{\sigma^{2}}| \boldsymbol{\gamma} \sim \Gamma\left(\frac{\nu}{2}, \frac{ss}{2}\right),  $$

where $\Gamma \left (\frac {\nu }{2}, \frac {ss}{2}\right)$ represents a gamma distribution with mean $\frac {\nu }{ss}$ and variance $\frac {\nu }{ss^{2}}$. We retained the default values of *ν*=0.01 and $ss = 0.5 \cdot {s}_{y}^{2}$ from the *BoomSpikeSlab* R package [43]. ${s}_{y}^{2}$ is the standard deviation of the response. We set the expected model size, *k*, to the default value of 1 in the variant termed BMA. For the variant termed BMAC, we estimated *k* with five-fold cross-validation to minimize the MSE. This enabled the BMAC variant to adapt to the inherent sparsity setting in the data.

The BMA procedure incorporates a MCMC algorithm that traverses a very large space of models to estimate the posterior distribution of the regression model parameters [44]. For our datasets, the running means of the selection indicators and the regression coefficients converged well before 10,000 MCMC iterations. Hence, we ran the MCMC algorithm for 10,000 iterations and discarded the initial 1000 iterations as burn-in. We then estimated the variable inclusion probabilities from the remaining 9000 iterations. The inclusion probability for the *i* ^*th*^ variable is the proportion of iterations (or draws) with non-zero regression coefficients

(16) $$ P(\gamma_{i}=1) = \frac{1}{D} \sum\limits_{d=1}^{D} 1_{\beta_{i} \ne 0}(M_{d}),  $$

where *D*=9000 is the total number of draws of model parameters from the posterior distribution, and *M*_*d*_ indicates the model parameters at iteration *d*.

### Variable selection

In high-dimensional settings, variable selection results in a sparse set of variables which provide the best explanation for the data. ENC performs variable selection by assigning the non-influential regression coefficients to zero. PS and PR directly compute the set of influential variables. For all the other approaches that generate variable inclusion probabilities, we applied a variable selection algorithm. In this algorithm, we first ranked the inclusion probabilities in decreasing order. We then computed the first order lagged differences between the ranked inclusion probabilities, identified the largest gap in this sequence, and selected the variables above this gap as influential. Suppose IP_*i*_,*i*∈{1,2,…*p*} denote the ranked inclusion probabilities, then the index that corresponds to the largest gap in inclusion probabilities is

(17) $$ \hat{i} = \mathop{\text{argmax}}\limits_{i}\left\{\text{IP}_{i} - \text{IP}_{i+1} \right\},  $$

and the set $\hat {\mathcal {V}}_{i} = \{v_{1}, v_{2}, \ldots v_{\hat {i}}\}$ is the chosen set of influential variables. We refer to this strategy as the lagged differences (LD) algorithm.

### Evaluation metrics

We evaluated the approaches on the basis of their ability to select the truly influential variables as well as their ability to rank these variables accurately.

#### Variable selection

**ROC curves and AUC score** The receiver operating characteristic (ROC) curve is a visual depiction of the variable selection performance of an approach across a range of thresholds on the inclusion probability. We constructed the ROC curve for each approach by plotting true positive rate (TPR) versus false positive rate (FPR) while sweeping across a sequence of 22 inclusion probability thresholds between 0.00 and 1.01 on the logarithmic scale. True positives (TP) are those variables which were correctly identified as influential by an approach. False positives (FP) are those which were incorrectly identified as influential by an approach. TPR and FPR are defined as follows

(18) $$ TPR = \frac{TP} {|\mathcal{V}_{i}|} \,\, FPR = \frac{FP}{p-|\mathcal{V}_{i}|},  $$

where *p* is the total number of variables and $\mathcal {V}_{i}$ is the set of all influential variables in the simulated data. In constructing the ROC curve for any given approach, variables with inclusion probabilities above a given threshold were counted as influential variables selected at that threshold. We also computed the area under the ROC curve (AUC) score for each of the approaches. The AUC is a single metric that quantifies the performance of any given approach across the complete range of inclusion probability thresholds [47]. Both the ROC curve and the AUC metric allowed us to assess the performance of approaches that generated inclusion probabilities for variables but did not perform variable selection. Therefore, these metrics evaluated model performance independent of any particular algorithm for variable selection.

##### F-score

The F-score is a single metric that measures the accuracy of variable selection with respect to the truly influential variables. It is the harmonic mean of precision and recall and is given by

(19) $$ \begin{aligned} \text{Precision} &= \frac{\text{TP}}{\text{TP+FP}}\\ \text{Recall} &= \frac{TP} {|\mathcal{V}_{i}|}\\ \text{F-score} &= 2 \left(\frac{\text{Precision} \times \text{Recall}}{\text{Precision} + \text{Recall}} \right). \end{aligned}  $$

#### Variable ranking

We measured the Spearman’s rank correlation coefficient between the simulation-assigned ranks *r*=(*r*_1_,*r*_2_,…*r*_*p*_) and the observed ranks $\hat {r} = (\hat {r}_{1}, \hat {r}_{2}, \ldots \hat {r}_{p})$ of the variables. For variable *i*, *r*_*i*_ was based on the absolute value of its regression coefficient while $\hat {r}_{i}$ was based on its inclusion probability from a given approach.

The Spearman’s rank correlation between *r* and $\hat {r}$ is specified by

(20) $$ \rho = \frac{\sum_{i=1}^{p} (r_{i} - \bar{r}) (\hat{r}_{i} - \bar{\hat{r}})} { \sqrt {\sum_{i=1}^{p} (r_{i} - \bar{r})^{2} \sum_{i=1}^{p} (\hat{r}_{i} - \bar{\hat{r}})^{2} }},  $$

where $\bar {r}$ and $\bar {\hat {r}}$ are the mean ranks of the true regression coefficients and the inclusion probabilities, respectively.

## Results and discussion

### Receiver operating characteristic (ROC)

**Figure 2:**
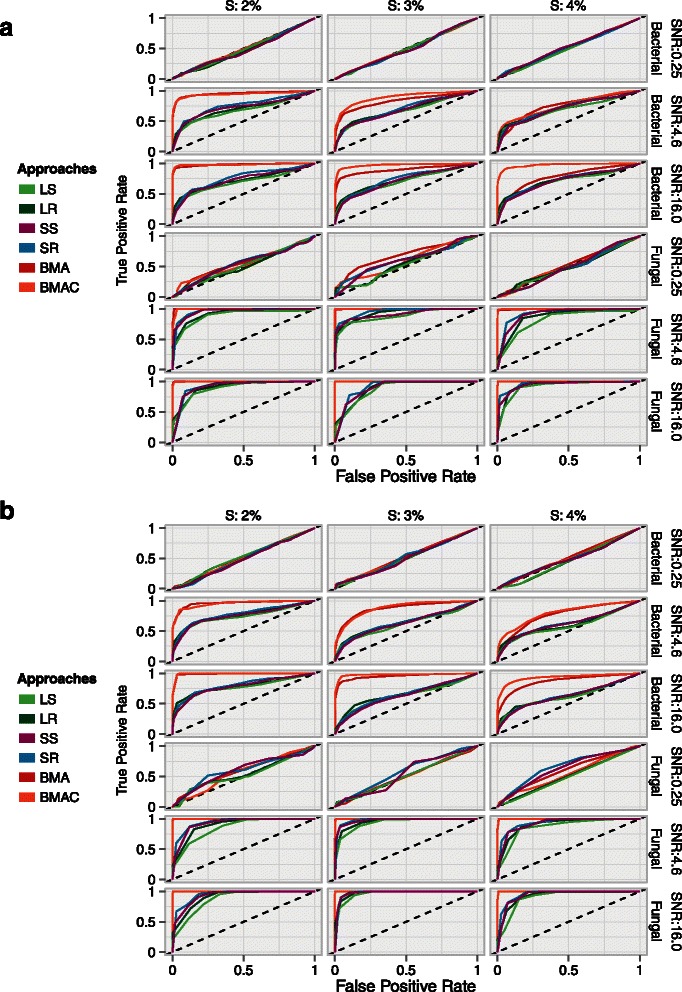
**Receiver operating characteristic (ROC) curves.** Mean ROC curves across 130 simulations are shown for approaches that do not perform variable selection. An ideal ROC has a high TPR and a low FPR over a wide range of thresholds. *S* denotes the sparsity setting or the proportion of influential variables in the data. The diagonal dashed line represents random variable selection performance. **a**. *β*=±1 and **b**. ${\beta \in \mathcal {U}}$.

Figure 2 shows the mean ROC curves, computed over 130 simulations, for approaches that assign inclusion probabilities to variables without performing variable selection. Good performance is characterized by high TPR and low FPR over a wide range of inclusion probability thresholds. The diagonal line on the plots represents random variable selection performance. ROC curves below the diagonal indicate poor performance while those above have better than random performance.

Overall, BMA variants consistently outperformed all the other approaches. Performance of all approaches deteriorated in high dimensions and with increasing number of influential variables, demonstrating that all ensembles had uniform difficulty in variable selection within less-sparse settings. Indeed, the setting with the highest percentage (4%) of influential variables and largest regression coefficients (*β*=±1) presented the most challenging scenario. The performance gap between BMA and the other approaches decreased in this case.

The relatively strong performance of all approaches in the low-dimensional fungal setting was most likely due to the smaller space of variable configurations. In most scenarios, SS and SR performed better than LS and LR approaches, illustrating the advantage of moving from a single *λ*_*optimal*_ to a larger model space consisting of a grid *λ*∈*Λ*.

At higher SNRs, BMA with cross-validated expected model size (BMAC) performed better than the other approaches. We also note that higher SNRs were uniformly associated with better performance.

### Area under the ROC curve (AUC)

**Figure 3:**
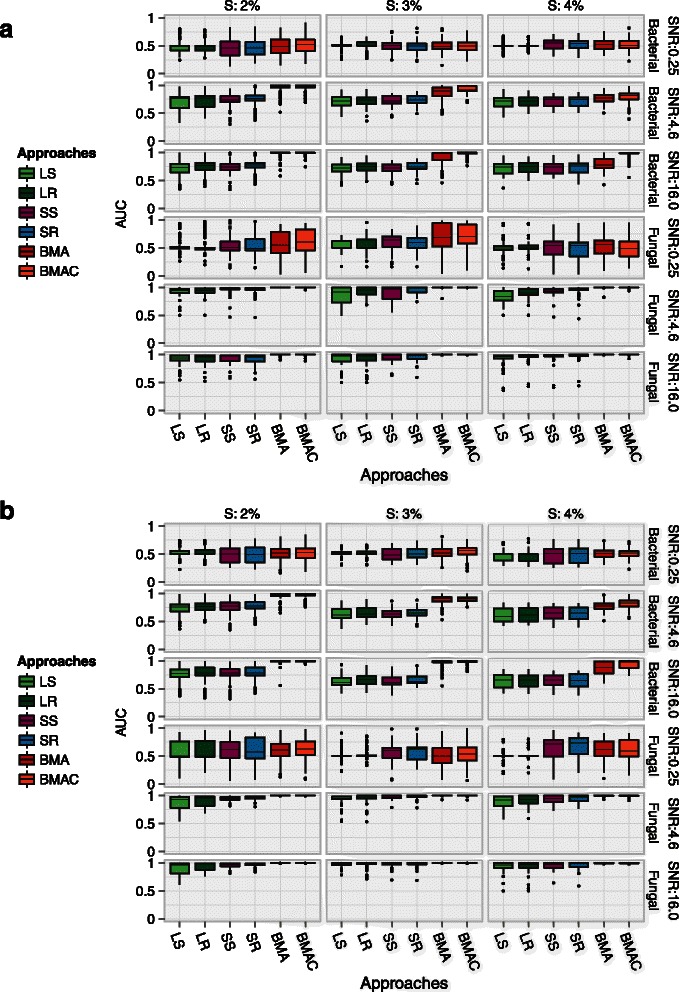
**Variation in area under the ROC curve (AUC) across 130 simulations are shown for approaches that do not perform variable selection.** Points beyond the end of the whiskers denote outliers. An AUC of 1.0 is ideal. *S* denotes the sparsity setting or the proportion of influential variables in the data. **a**. *β*=±1 and **b**. ${\beta \in \mathcal {U}}$.

Figure 3 shows the variability in AUC across 130 simulations for the approaches that assign inclusion probabilities. The corresponding median AUCs are shown in Additional file 1. An AUC of 1.0 is ideal and corresponds to a scenario where an approach consistently ranks influential variables higher than non-influential variables. In our simulations, the trends in AUC mirrored the patterns in the ROC curves. BMA variants and specifically, BMAC, obtained the highest AUC along with the lowest variability, showing the advantages of exploring the largest model space among all approaches.

While BMA variants had a near perfect AUC in the low-dimensional fungal setting, the AUCs for non-BMA approaches were also higher in this setting. Although SS and SR had AUCs similar to BMA in the fungal setting, they had a much higher variability. The resampling variants, LR and SR, yielded small but consistent improvements in median AUC relative to their subsampling counterparts in the high-dimensional setting.

The lowest SNRs resulted in the lowest AUCs and the highest variability. Since SNRs of <0.25 are not representative of our experimental datasets and in addition, yielded uniformly poor performance across all ensembles, we report results only for medium and high SNR situations in the evaluations that follow.

### F-score

**Figure 4:**
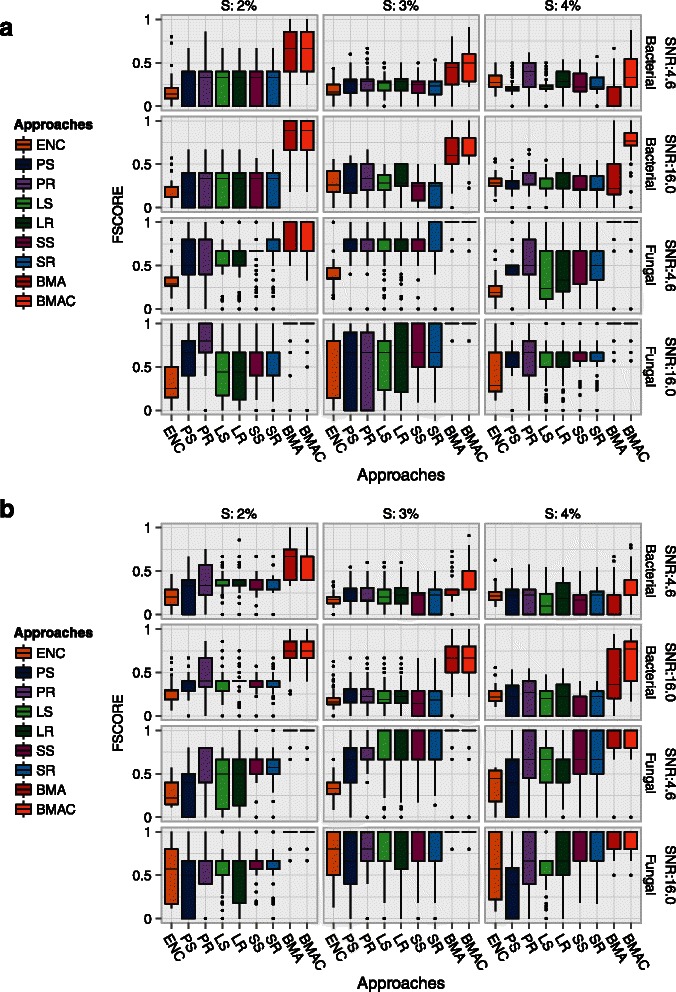
**Variation in F-score across 130 simulations.** Points beyond the end of the whiskers denote outliers. An F-score of 1.0 is ideal. The LD algorithm was used to select variables for approaches that do not perform variable selection. SNR=0.25 is not shown. *S* denotes the sparsity setting or the proportion of influential variables in the data. ***a***. *β*=±1 and ***b***. ${\beta \in \mathcal {U}}$.

Higher values of the F-score indicate a good balance between precision and recall. Figure 4 shows the variation in F-scores across 130 simulation runs for all approaches while Additional file 1 shows the medians. For methods that did not perform variable selection, we applied the inclusion probability threshold from the LD algorithm to select the top influential variables.

BMAC outperformed all the other approaches across all settings. BMA was a close second, however, its performance deteriorated at lower sparsity settings. As was pointed out by one of our referees, this finding shows the benefits of cross-validating the *expected model size* within BMAC, enabling it to adapt better to the sparsity in the data. The PR algorithm performed as well as BMAC in the low-sparsity and medium SNR settings within high dimensions and in the presence of large regression coefficients (*β*=±1). This would suggest that resampling conferred a distinct advantage over subsampling, enabling PR to perform well despite its small model space.

In lower dimensions, BMA approaches, with the largest ensembles, outperformed all other approaches across all SNR settings. SS and SR performed similar to BMA in a number of low-dimensional fungal settings but the variability in their performance was substantially higher.

### Spearman’s rank correlation

**Figure 5:**
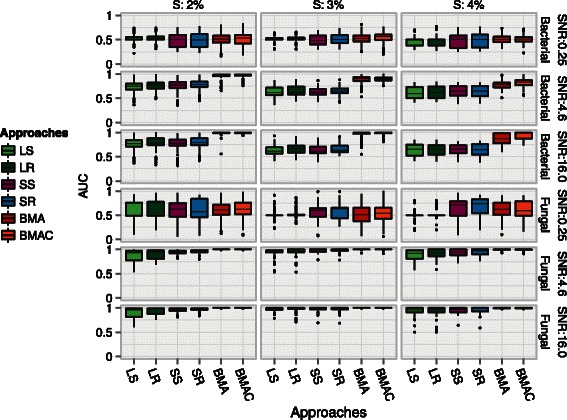
**Variation in Spearman’s rank correlation (** $\beta \in \boldsymbol{\mathcal {U}}$ **) across 130 simulations are shown for approaches that do not perform variable selection.** Points beyond the end of the whiskers denote outliers. A correlation of 1.0 is ideal. Higher correlation indicates that the approach is able to capture the original variable rank more accurately. SNR=0.25 is not shown. *S* denotes the sparsity setting or the proportion of influential variables in the data.

Figure 5 shows the variable ranking performance measured using Spearman’s rank correlation between the estimated and true variable ranks for each of the approaches for $\beta \in \mathcal {U}$. The corresponding median correlations are shown in Additional file 1. Overall, the rank correlation decreased with an increase in dimensionality. BMA approaches showed higher correlation and lower variability relative to the other approaches in high dimensions. However, all approaches performed similarly in low dimensions.

### Additional simulations

We performed two additional simulations to address points raised by our referees.

#### Performance without log-transformation

In this simulation, we examined the performance of all ensembles on relative abundance data that were not log-transformed. Data without log-transformation showed similar trends in performance as log-transformed data (Additional file 2). However, the number of outliers increased across all metrics in the data without log-transformation. This suggests that log-transformation of relative abundances decreases variability in the performance of ensembles.

#### Weighted average of inclusion probabilities

We also performed simulations to compare SS and SR with their weighted counterparts, SSW and SRW, computed using Equation 9. Overall, the performance of the weighted approach was not consistently different from the unweighted approach (Additional file 3). However, there were minor increases in the median performance of the weighted approach in high-SNR settings.

### Application to mouse microbiome data

We now illustrate the application of the ensembles (Table 2) to identify influential variables in our experimental mouse microbiome data collected from the terminal ileum. Briefly, the gastrointestinal tract is an ecological niche for both bacterial and fungal flora. By depleting the competing bacterial microbiota, antibiotics create vacancies in the niche that co-existing fungi can repopulate. However, a high level of colonization by a single fungal species such as *C. albicans* is an unfavorable clinical outcome [48], especially in immunosuppressed and dysbiotic patient populations [49-51]. We therefore designed our mouse microbiome study to examine the factors influencing *C. albicans* colonization of the gastrointestinal tract following initiation of antibiotic treatment.

The experimental design of our mouse microbiome study is summarized in Figure 1 and Table 1. Following exposure to antibiotics and *C. albicans*, we profiled the microbial communities in the mouse gut by amplifying and sequencing the taxonomically discriminant bacterial 16S rDNA [52] from the V3-V5 variable region and the fungal ITS rDNA [53]. Using the YAP bioinformatic workflow [54], we obtained taxonomic assignments that reach genus-level resolution for both bacteria and fungi. Assignments unclassified at the genus-level were annotated with the prefix *UC*. The median number of reads per sample was 3500 for bacterial 16S and 2000 for fungal ITS regions. We built two linear regression models to assess the association of bacteria and fungi with the level of *C. albicans* colonization. We estimated the bacterial models from 30 samples and the fungal models from 36 samples. Both the bacterial and fungal models were specified as follows

(21) $$ Y = {\boldsymbol X}_{g}\boldsymbol{\beta}_{g} + {\boldsymbol X}_{c}\boldsymbol{\beta}_{c} + {\boldsymbol X}_{a}\boldsymbol{\beta}_{a} + \boldsymbol{\epsilon},  $$

where *Y* is the colonization level measured in $log \left (\frac {\text {Colony forming units (CFU)}}{\text {grams of fecal matter}}\right)$, ***X***_*g*_ is a vector of log relative abundances of sequences assigned to bacterial or fungal genera, ***X***_*c*_ is a vector of log normalized mRNA expression levels of cytokines, ***X***_*a*_ is a vector of 5 binary variables indicating antibiotic treatment (vancomycin or PSG: penicillin, streptomycin and gentamicin) for a span of 7 or 21 days and exposure to *C. albicans* on the 7th day, and ***ε*** is the residual error in the model.

Since the microbial proportions sum to one, the proportion of any given genus is known given the proportions of all the other genera. To avoid this redundancy, we could exclude the proportion of any one reference genus. However, doing so would mean we are unable to obtain inclusion probability for this genus. We thus selected two reference genera with the highest abundances across all samples and repeated the model building procedure twice, each time excluding one of the two reference genera from ***X***_*g*_. We estimated the final set of selected variables and inclusion probabilities for all genera by averaging across these two models.

We applied each of the approaches (Table 2) from the simulation study to this data to discover the microbiome-immune-antibiotic signatures associated with the level of *C. albicans* colonization. The inclusion probability for any variable reflects its importance to the response. For methods that do not perform variable selection, we used the LD algorithm to derive an inclusion probability threshold for identifying the top influential variables. Since ENC, PS and PR directly provide us with lists of influential variables and do not assign inclusion probabilities, we annotated the inclusion probabilities for these influential variables as 100%.

#### Mouse microbiome findings

**Figure 6:**
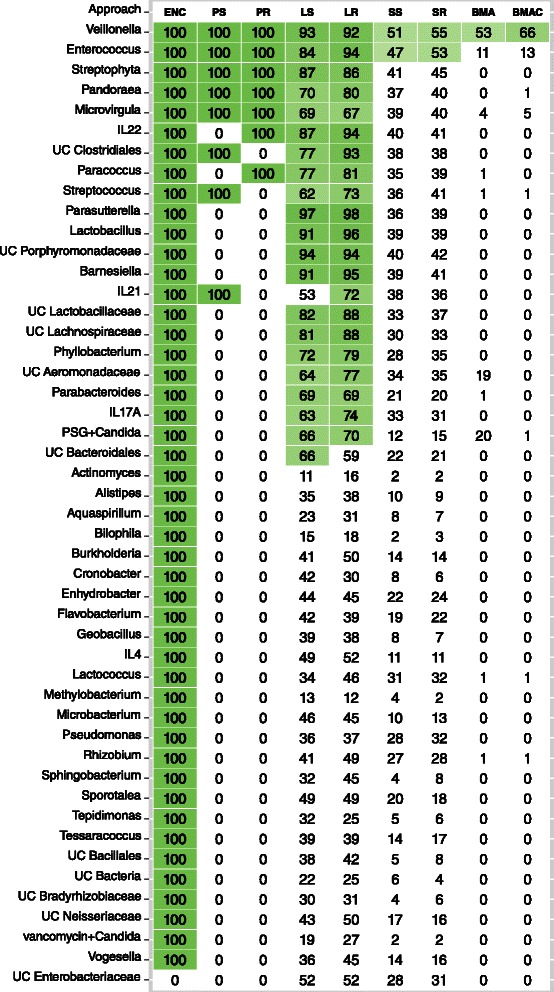
***Candida*** **colonizatin: Influential variables in the bacterial model.** Variables selected in each of the modeling approaches are highlighted in green. The LD algorithm was utilized for selecting variables for approaches that do not perform variable selection. Values in the cells are inclusion probabilities expressed in %. ENC, PS and PR do not generate inclusion probabilities. In these models, an inclusion probability of 100% indicates that the variable was selected as influential.

Figure 6 shows the bacteria and cytokines identified as influential by the approaches along with their inclusion probabilities (in %). ENC showed the least sparsity and selected the maximum number of bacteria and host immune cytokines. Although *Veillonella* was identified as an influential variable in all approaches, only BMA, BMAC, SS and SR assigned it the highest inclusion probability. However, unlike SS and SR, the difference in the inclusion probabilities of *Veillonella* and the other influential variables was much larger in the BMA models.

**Figure 7:**
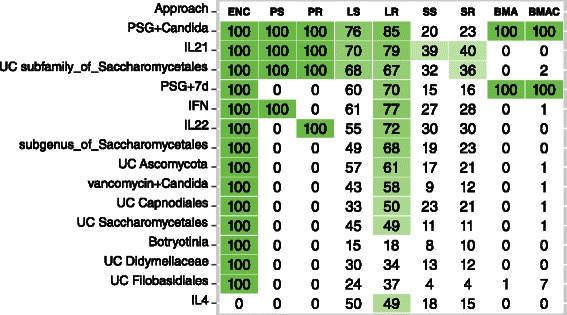
***Candida*** **colonization: Influential variables in the fungal model.** Variables selected in each of the modeling approaches are highlighted in green. The LD algorithm was utilized for selecting variables for approaches that do not perform variable selection. Values in the cells are inclusion probabilities expressed in %. ENC, PS and PR do not generate inclusion probabilities. In these models, an inclusion probability of 100% indicates that the variable was selected as influential.

Figure 7 shows results from the fungal model. Treatment with PSG for a span of (a) 7 days, and (b) 21 days along with exposure to *C. albicans* were both assigned very high inclusion probabilities by BMA and BMAC. Unlike the bacterial model, the highest-ranking variables in SS and SR were different from those in BMA approaches. PR and PS shared common variables with both SS and SR and the BMA approaches. The other single *λ* approaches (LR, LS) showed some similarities with SS and SR, however, their inclusion probability assignments were substantially different. ENC showed a lack of sparsity analogous to the bacterial models.

Viewed together, findings from the bacterial and fungal models indicate that the type of antibiotic, span of exposure to the antibiotic and specific antibiotic-induced changes in the bacterial microbiota substantially influenced the levels of *C. albicans* colonization. The concurrent effects of the host immune cytokines and the co-existing fungal microbiota on the level of *C. albicans* colonization were weaker. These findings are promising since they suggest that tailoring antibiotic regimens as well as concomitant modulation of the microbiota during antibiotic administration could be employed to reduce opportunistic *C. albicans* colonization.

#### Comparison of approaches

**Figure 8:**
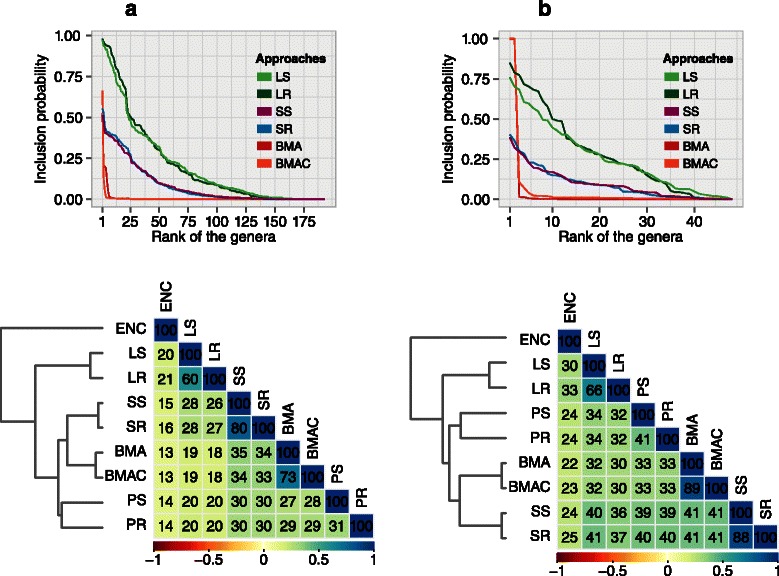
**Comparison of inclusion probability profiles across all approaches.** The top panels show the sorted inclusion probabilities plotted against the rank of the variables in the model. The bottom panels show the pairwise similarities between the inclusion probability profiles of all the approaches. The similarity between any pair of approaches *i* and *j* is given as $S_{\textit {ij}}=\frac {1}{1+D_{\textit {ij}}}$, where *D*_*ij*_ is the Euclidean distance between the inclusion probability vectors of approaches *i* and *j*. We generated the dendrogram using agglomerative clustering with the centroid linkage method. We then ordered the correlogram using the order of models generated in the dendrogram. **a**. Bacterial models, **b**. Fungal models.

To further examine similarities and differences in the workings of each of the modeling approaches, we plotted the inclusion probability versus rank for all variables across the ensembles (Figure 8). In both high and low-dimensional settings, the approaches separated out into three distinct groups. In the first group that included BMA and BMAC, inclusion probabilities showed the steepest decay. In the second, comprising the SS and SR, the decay was more gradual. The single *λ* approaches constituted the third group. Here the rate of decay was similar to SS and SR, however the dynamic range of inclusion probabilities was larger. The sharper decay in the BMA methods indicates a more discriminative ranking that allows a distinct separation of the influential variables from the non-influential ones.

On clustering the approaches based on Euclidean distance between the inclusion probability vectors, the single *λ* approaches were further away from PS, PR, SS, SR and the BMA approaches, indicating their divergent performance. The proximity of SS and SR to BMA approaches suggests that SS and SR, computed over a much larger number of subsamples or resamples, could potentially approach the performance profile of BMA.

## Conclusions

Microbiome studies constitute a recently developed area of research that incorporate NGS to profile communities of diverse microbes residing in a variety of ecosystems. As a result, microbiome data shares many of the characteristics of NGS data that make it challenging to analyze. These challenges arise from high-dimensionality, limited sample sizes, multicollinearity within covariables, modest SNRs and a very large space of models. Ensemble modeling is able to provide a comprehensive solution to these analytical challenges. However, except for non-linear ensembles such as random forests, ensemble modeling has not been adopted widely in microbiome research.

Our goal in this paper was to familiarize investigators in the field with the characteristics of both frequentist and Bayesian ensemble-based regression approaches and present an evaluation of these approaches on realistic microbiome data. We also introduce a simulation framework that can be built from any experimental microbiome dataset to closely mimic the characteristics of real data. We demonstrate the usefulness of this framework in evaluating analytical approaches using a suite of metrics that assess various facets of modeling performance. Unlike other simulation strategies [4,16] that sample covariates from parametric distributions, we directly utilize the covariance structure, SNR and the dimensions inherent in our experimental mouse data for our simulation. Even though parametric simulation approaches enable generation of arbitrary number of samples, we expect the findings from our simulation-based evaluation to translate better to experimental datasets. Our implementation, in the R statistical language, is freely available from *GitHub* [30], making it convenient for researchers in the microbiome research community to adopt and apply our evaluation methodology to their own datasets without the need for expensive ground truth annotations.

We found that the Bayesian approaches had several favorable attributes that distinguished them from the other ensembles we evaluated. In realistic settings with medium SNRs and uniformly distributed coefficients for influential variables ($\beta \in \mathcal {U}$), they performed consistently better or at par with frequentist approaches depending on the number of influential variables. Furthermore, they showed substantially less variation across replications. Selecting the optimal *expected model size* via cross-validation (BMAC) improved performance in more challenging high-dimensional settings with a large number of influential variables. PR, the resampling variant of the original stability selection algorithm [22] performed as well as BMAC in these scenarios, highlighting its ability to perform effective variable selection when there are several variables with large regression coefficients. On the whole, the resampling variants had small but consistent performance gains over their subsampling counterparts, suggesting that resampling was able to create an improved model space relative to subsampling. Even though SS, SR, PS and PR, the frequentist ensembles, performed reasonably well at lower dimensions, they exhibited substantially more variability in their performance. Our findings thus enabled us to pick BMAC for our experimental setting with modest sample sizes and high-dimensionality in measurements.

We note that BMAC uses the data for both estimating a hyperparameter of the prior distribution and for computing the posterior distribution. It is therefore not a full Bayesian approach. An ideal alternative would be to employ a full Bayesian model that includes a hyperprior for the prior inclusion probabilities [55]. While our work has systematically evaluated a powerful set of sparse linear regression modeling methods that average across large model spaces in the context of continuous response variables, a future direction would be to extend these evaluations for analyzing categorical responses and time-to-event outcomes that are prevalent in clinical settings. These models include logistic, multinomial, and survival regression.

Our analysis could have been performed at the higher taxonomic levels of phylum, class, order and family. However, we selected the genus-level primarily because it provided the closest taxonomic resolution to our response (*C. albicans* colonization). A limitation of our approach, which is shared by other analytical approaches such as [4,16,17], is the assumption that taxonomic assignment for all organisms at all phylogenetic levels is equally accurate. As a result, covariables are treated alike regardless of classification accuracy. However in practice, classification error could vary across phylogenetic branches as well as taxonomic levels and depends on factors such as the quality of annotated taxonomic databases as well as the other parameters of underlying bioinformatic algorithms [56]. A crucial avenue of future research would be to integrate information from models constructed from taxonomic assignments at varying classification accuracies.

Another area that we have not explored in this work is the inclusion of interactions and variable sub-structures within the high-dimensional regression framework. In the microbiome setting, this would involve several higher-order interaction terms among taxa. Many such symbiotic and antagonistic relationships are known to exist among taxa and cytokines but are hard to assess in analytical settings. Including these interactions is computationally challenging because they would increase the model space exponentially. Recent developments in statistical algorithms that efficiently explore this additional complex model space [57,58] hold promise for making this problem more tractable at the scale of microbiome data. In the context of biological interpretations, it could be useful to integrate variable clustering with ensemble-based regression in a framework similar to the one proposed in [59] to obtain a greater understanding of the most relevant dynamics and relationships in the community.

In conclusion, as microbiome studies evolve towards translational settings, analysts are likely to face challenges in selecting appropriate modeling strategies that yield consistent and stable performance with low variability [60]. Therefore, we expect that our research will provide both insights for choosing among ensemble methods and an evaluation framework critical for making an objective selection.

## Additional files

Additional file 1
**Median values of performance metrics (median_scores.pdf).** Figures showing median values of performance metrics presented as boxplots in the main paper.

Additional file 2
**Performance without log-transformation (no_log_transformation.pdf).** Figures showing performance of all the approaches on data without log-transformations.

Additional file 3
**Performance with weighted inclusion probabilities (weighted_ip.pdf).** Figures showing performance of SS, SR, SSW and SRW approaches.

## Abbreviations

AUC: Area under the ROC curve
BMA: Bayesian model averaging
BMAC: Bayesian model averaging with cross-validation for expected model size
*Bolasso*: Bootstrap-enhanced LASSO
CFU: Colony forming units
CV: Cross-validation
EMS: Expected model size
ENC: Elastic net with cross-validation
FP: False positives
FPR: False positive rate
GAPDH: Glyceraldehyde-3-Phosphate Dehydrogenase
GI: Gastrointestinal
ITS: Internal transcribed spacer
LARS: Least angle regression
LASSO: Least absolute shrinkage and selection operator
LD: Lagged differences
LR: Stability selection on resamples using *λ*_*ENC*_
LS: Stability selection on subsamples using *λ*_*ENC*_
MB: Meinshausen &
Bühlmann; MCMC: Markov chain Monte Carlo
MSE: Mean squared error
NGS: Next-generation sequencing
NIH: National Institutes of Health
PR: Stability selection on resamples minimizing expected number of false positives
PS: Stability selection on subsamples minimizing expected number of false positives
PSG: penicillin, streptomycin, gentamicin
ROC: Receiver operating characteristic
RSS: Residual sum of squares
SNR: Signal to noise ratio
SR: Stability selection on resamples using unweighted average inclusion probabilities across *λ*∈*Λ*
SRW: Stability selection on resamples using weighted average inclusion probabilities across *λ*∈*Λ*
SS: Stability selection on subsamples using unweighted average inclusion probabilities across *λ*∈*Λ*
SSW: Stability selection on subsamples using weighted average inclusion probabilities across *λ*∈*Λ*
TP: True positives
TPR: True positive rate
UC: Unclassified
