# Supplementary material for: A systematic evaluation of high-dimensional, ensemble-based regression for exploring large model spaces in microbiome analyses
Source: BMC Bioinformatics. 2015 Feb 1;16:31. doi: 10.1186/s12859-015-0467-6 (PMC4339743; doi:10.1186/s12859-015-0467-6)

Additional File 1
Median values of performance metrics.

For ease of interpretation, the following figures present the median values of performance metrics presented as boxplots in the main paper.

Figure 1: **Median AUC** across 130 simulations are shown for approaches that do not perform variable selection. An AUC of 1.0 is ideal. **a.** $\boldsymbol{\beta} = \pm\mathbf{1}$ and **b.** $\boldsymbol{\beta} \in \boldsymbol{\mathcal{U}}$

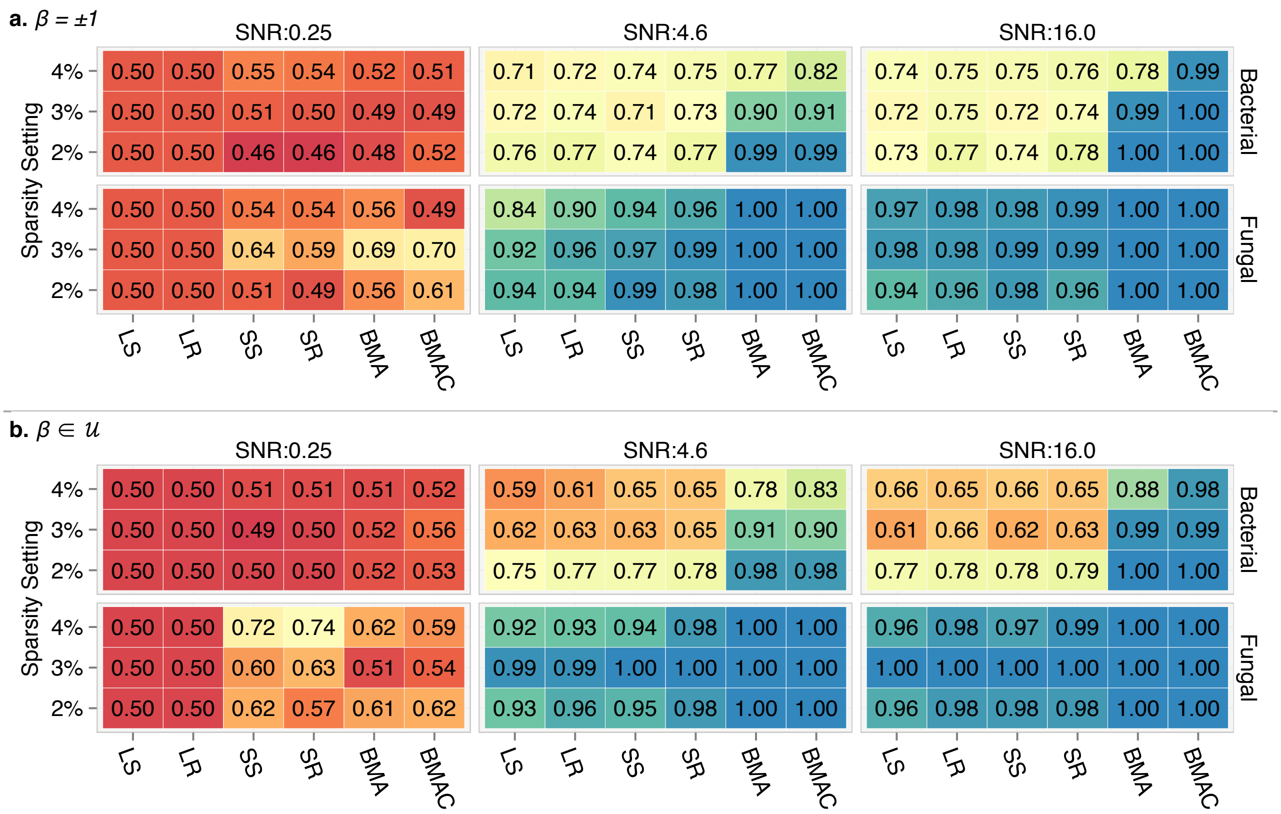

Figure 2: **Median F-score** across 130 simulations. An F-score of 1.0 is ideal. The LD algorithm was used to select variables for approaches that do not perform variable selection. SNR=0.25 is not shown. **a.** $\boldsymbol{\beta = \pm 1}$ and **b.** $\boldsymbol{\beta \in \mathcal{U}}$

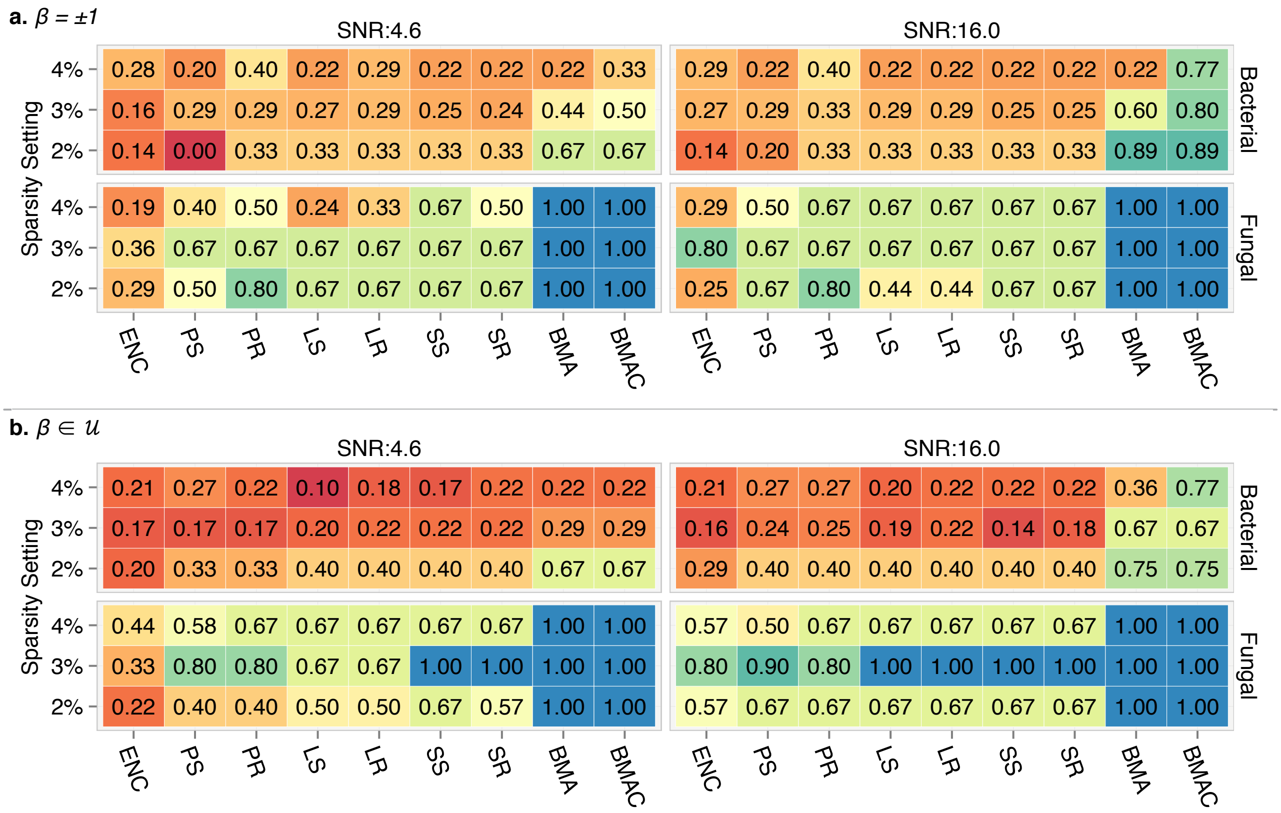

Figure 3: **Median Spearman's rank correlation** ($\boldsymbol{\beta \in \mathcal{U}}$) across 130 simulations are shown for approaches that do not perform variable selection. A correlation of 1.0 is ideal. Higher correlation indicates that the approach is able to capture the original variable rank more accurately. SNR=0.25 is not shown.

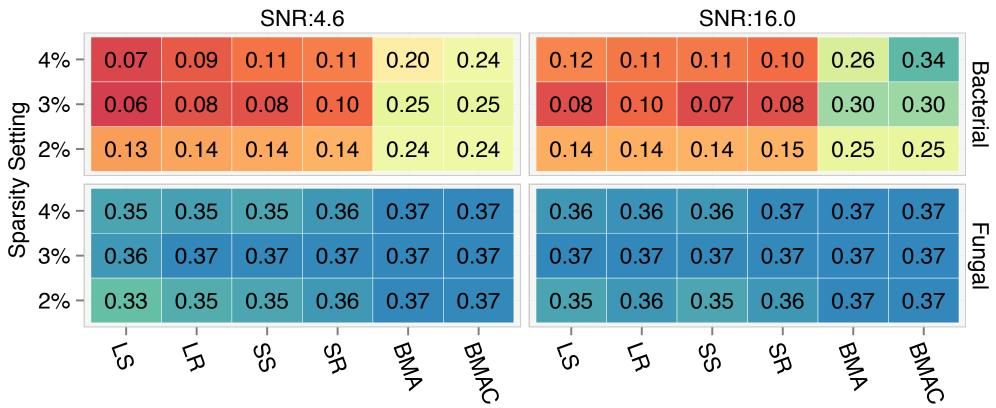

Supplement: Additional file 1 — Median values of performance metrics (median_scores.pdf). Figures showing median values of performance metrics presented as boxplots in the main paper. [file 12859_2015_467_MOESM1_ESM.pdf]
