# Supplementary material for: A systematic evaluation of high-dimensional, ensemble-based regression for exploring large model spaces in microbiome analyses
Source: BMC Bioinformatics. 2015 Feb 1;16:31. doi: 10.1186/s12859-015-0467-6 (PMC4339743; doi:10.1186/s12859-015-0467-6)

Additional File 2
Performance without log-transformation

Figure 1: **Variation in area under the ROC curve (AUC)** across 165 simulations are shown for approaches that do not perform variable selection. Points beyond the end of the whiskers denote outliers. An AUC of 1.0 is ideal. S denotes the sparsity setting or the proportion of influential variables in the data. **a.** $\boldsymbol{\beta = \pm 1}$ and **b.** $\boldsymbol{\beta \in \mathcal{U}}$

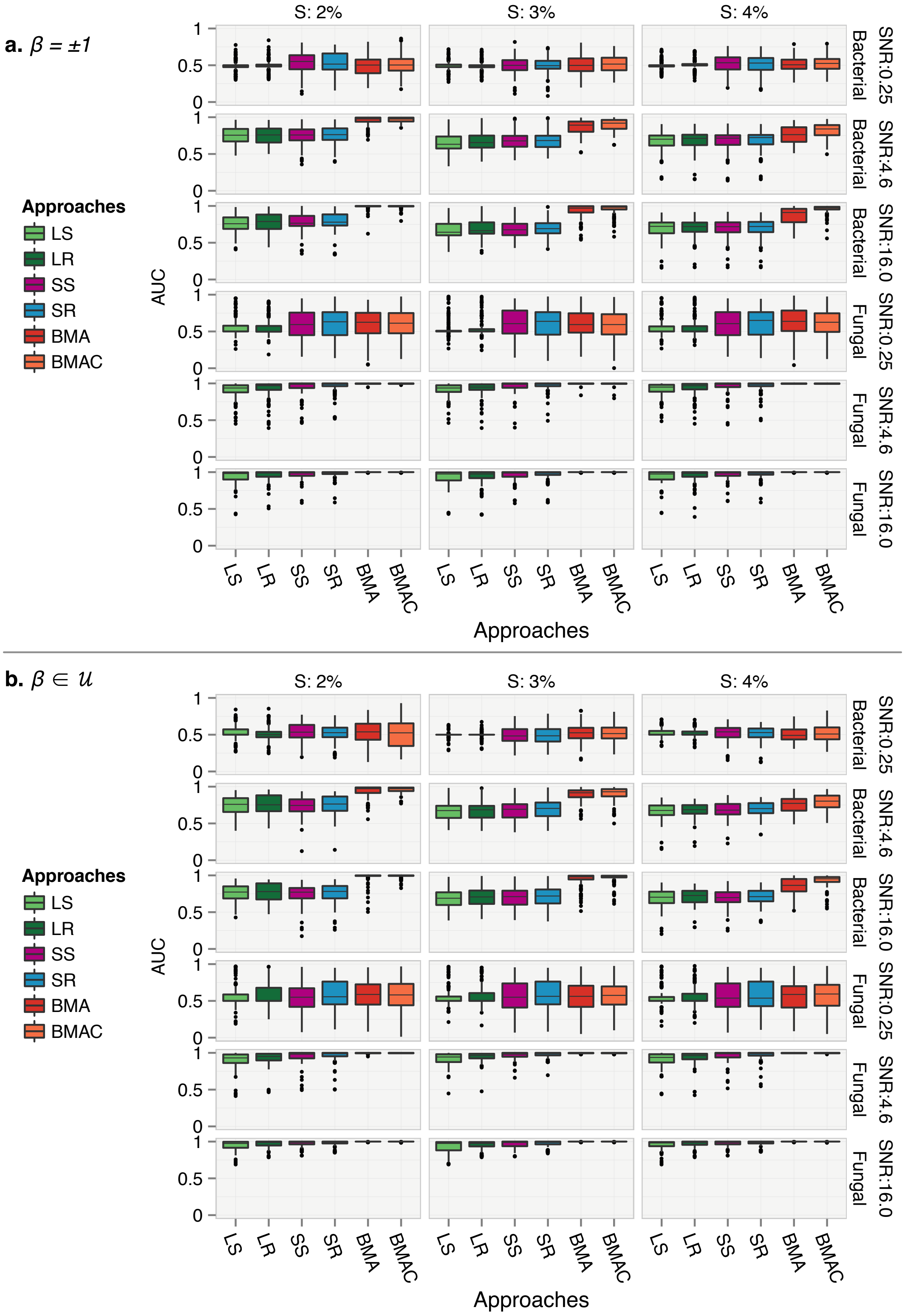

Figure 2: **Median AUC** across 165 simulations are shown for approaches that do not perform variable selection. An AUC of 1.0 is ideal. **a.** $\boldsymbol{\beta} = \pm\mathbf{1}$ and **b.** $\boldsymbol{\beta} \in \boldsymbol{\mathcal{U}}$

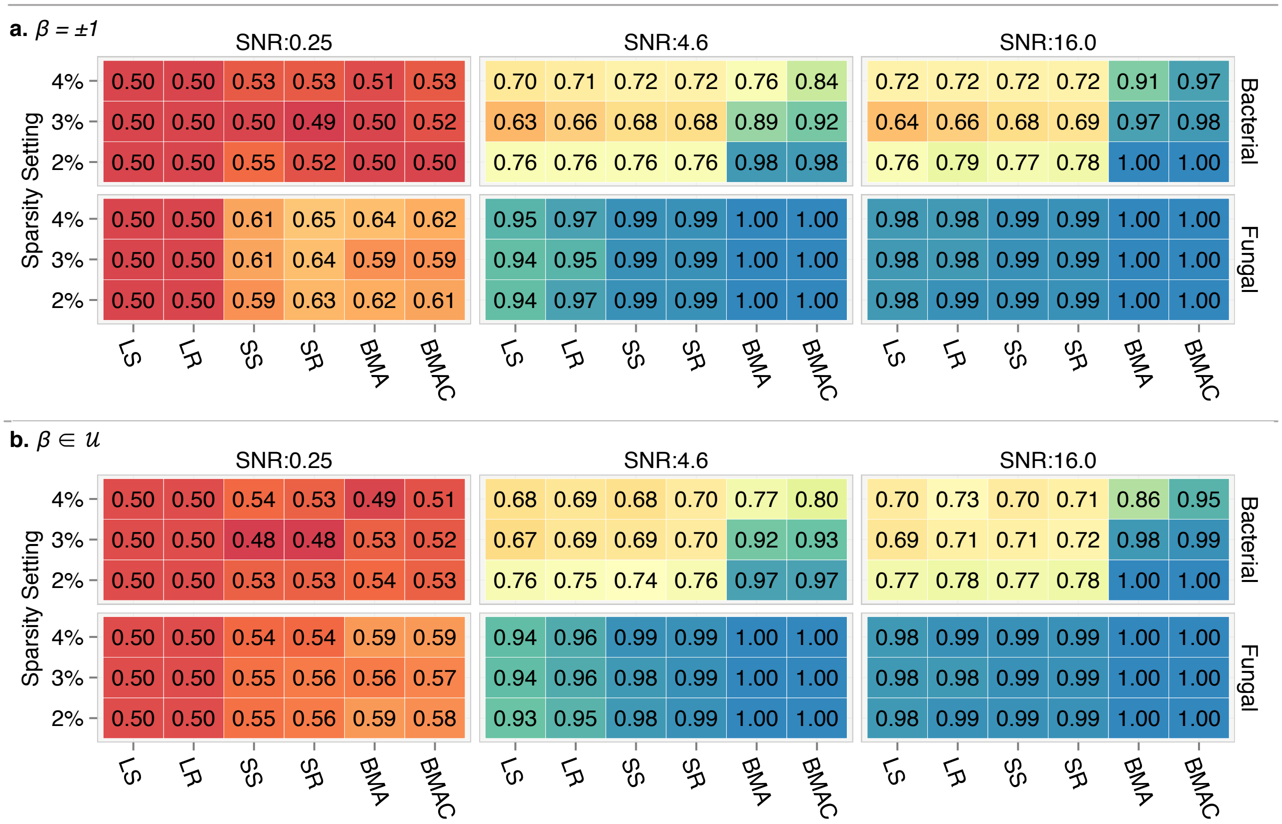

Figure 3: **Variation in F-score** across 165 simulations. Points beyond the end of the whiskers denote outliers. An F-score of 1.0 is ideal. The LD algorithm was used to select variables for approaches that do not perform variable selection. SNR=0.25 is not shown. S denotes the sparsity setting or the proportion of influential variables in the data. *a.* $\boldsymbol{\beta} = \pm\mathbf{1}$ and *b.* $\boldsymbol{\beta} \in \boldsymbol{\mathcal{U}}$

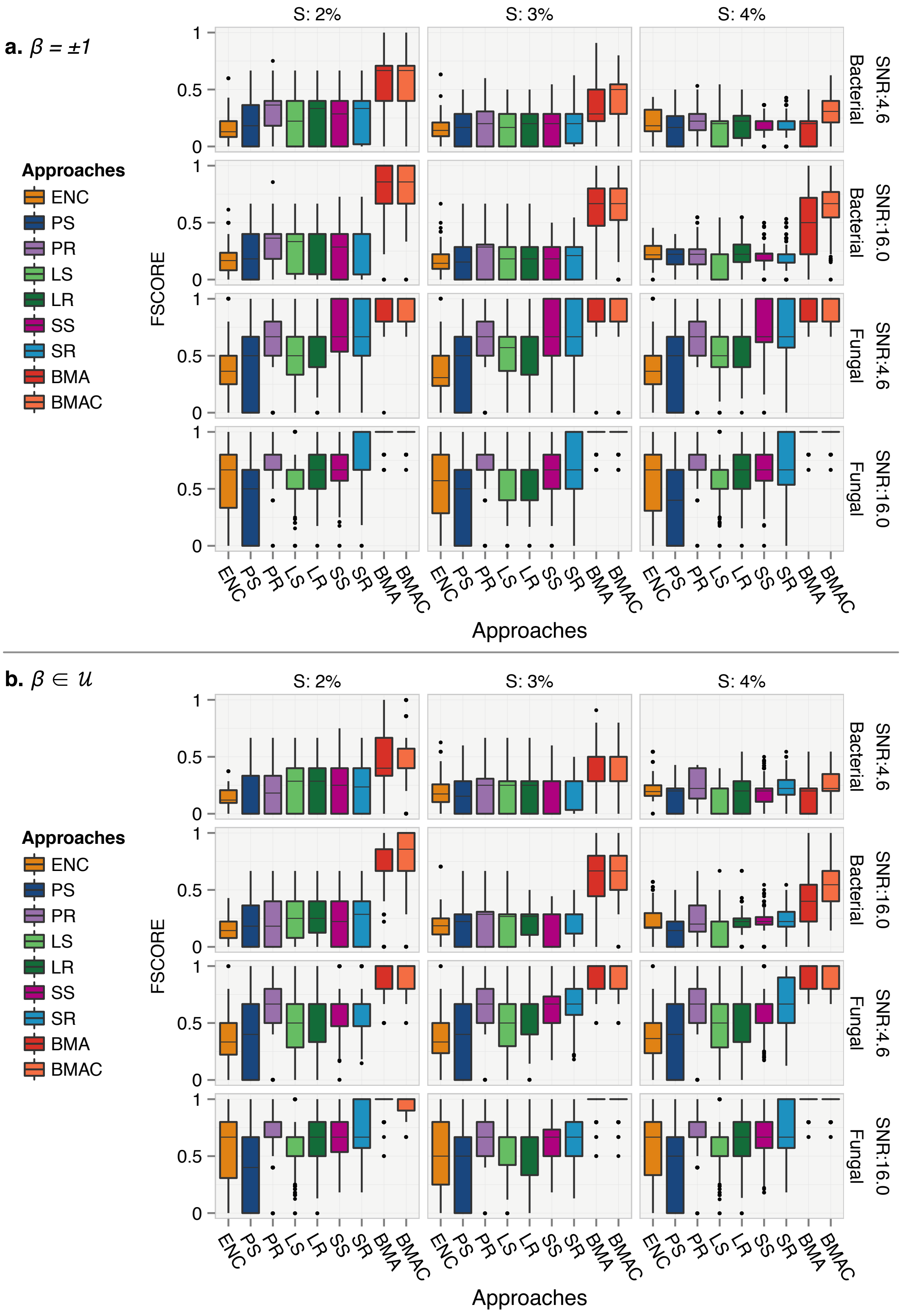

Figure 4: **Median F-score** across 165 simulations. An F-score of 1.0 is ideal. The LD algorithm was used to select variables for approaches that do not perform variable selection. SNR=0.25 is not shown. **a.** $\boldsymbol{\beta = \pm 1}$ and **b.** $\boldsymbol{\beta \in \mathcal{U}}$

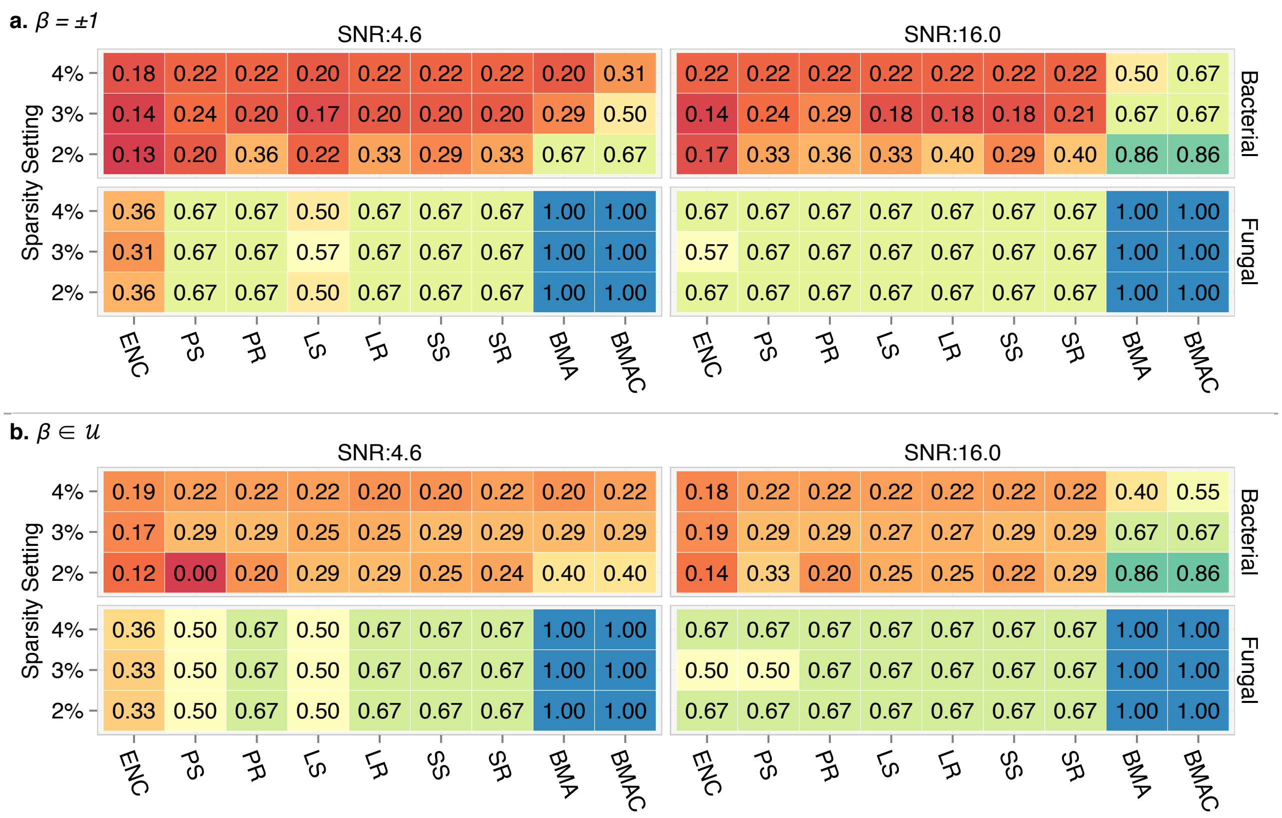

Figure 5: **Variation in Spearman's rank correlation ($\beta \in \mathcal{U}$)** across 165 simulations are shown for approaches that do not perform variable selection. Points beyond the end of the whiskers denote outliers. A correlation of 1.0 is ideal. Higher correlation indicates that the approach is able to capture the original variable rank more accurately. SNR=0.25 is not shown. S denotes the sparsity setting or the proportion of influential variables in the data.

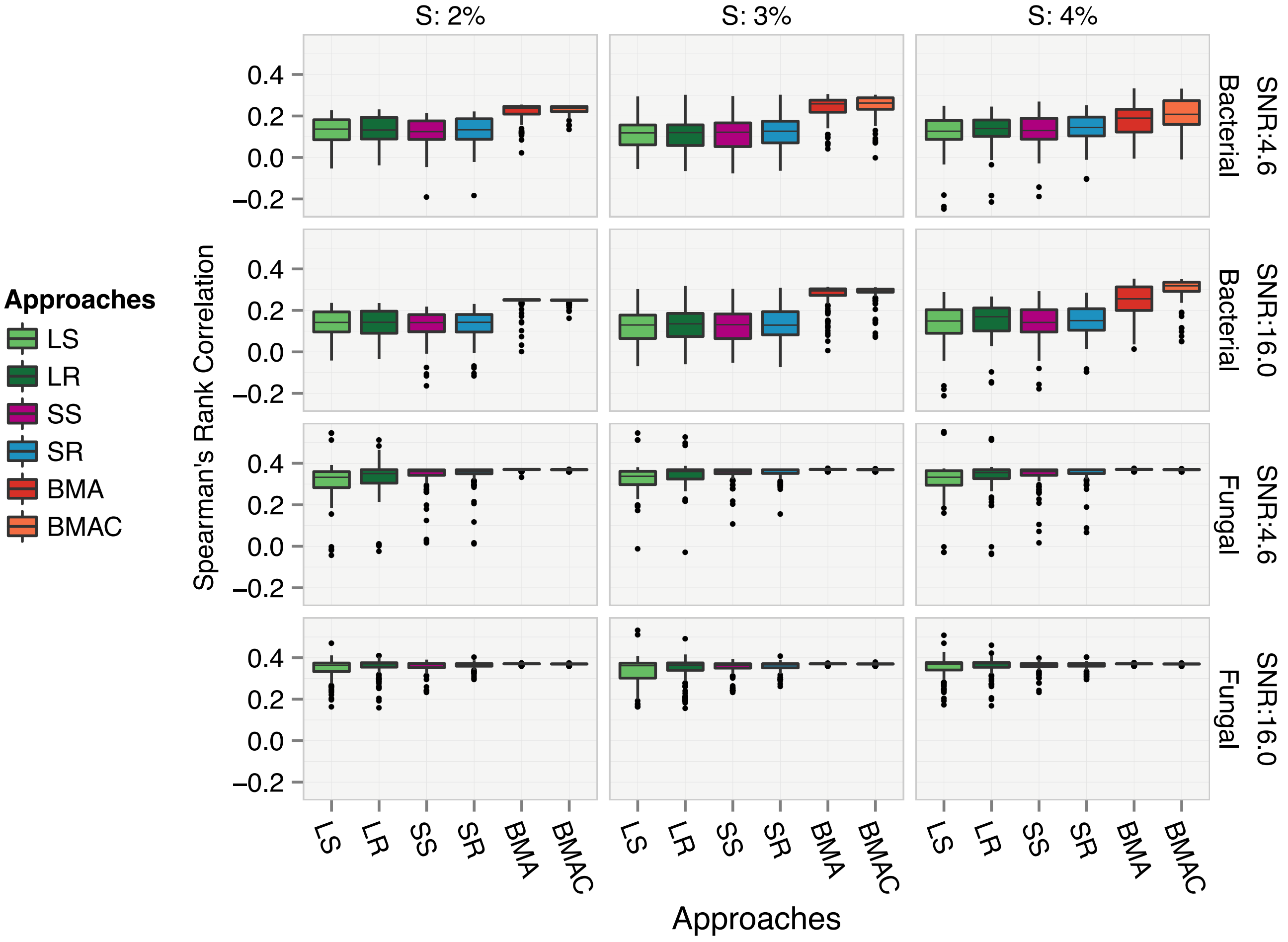

Figure 6: **Median Spearman's rank correlation ($\beta \in \mathcal{U}$)** across 165 simulations are shown for approaches that do not perform variable selection. A correlation of 1.0 is ideal. Higher correlation indicates that the approach is able to capture the original variable rank more accurately. SNR=0.25 is not shown.

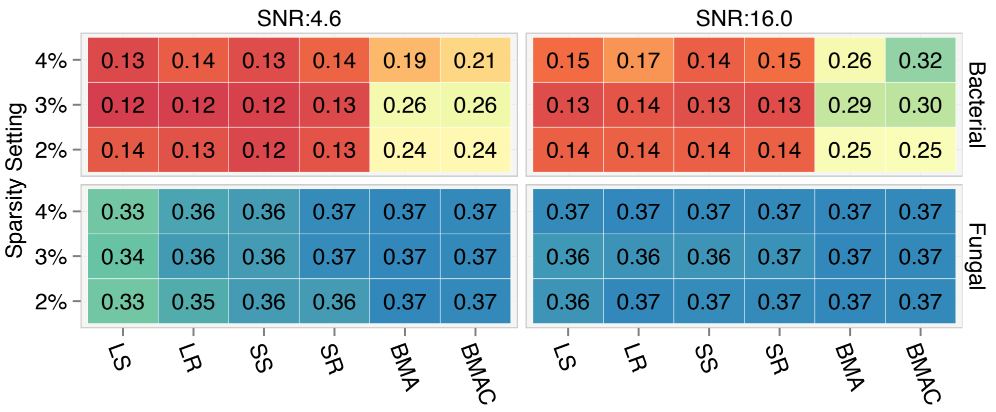

Supplement: Additional file 2 — Performance without log-transformation (no_log_transformation.pdf). Figures showing performance of all the approaches on data without log-transformations. [file 12859_2015_467_MOESM2_ESM.pdf]
